# Identification of B6T173 (ZmPrx35) as the prevailing peroxidase in highly insect-resistant maize (*Zea mays*, p84C3) kernels by activity-directed purification

**DOI:** 10.3389/fpls.2015.00670

**Published:** 2015-08-31

**Authors:** Laura M. López-Castillo, Janet A. I. López-Arciniega, Armando Guerrero-Rangel, Silvia Valdés-Rodríguez, Luis G. Brieba, Silverio García-Lara, Robert Winkler

**Affiliations:** ^1^Laboratory of Biochemical and Instrumental Analysis, Department of Biotechnology and Biochemistry, Cinvestav Unidad IrapuatoIrapuato, Mexico; ^2^Laboratorio Nacional de Genómica para la Biodiversidad, Unidad de Genómica Avanzada del Centro de Investigación y de Estudios Avanzados – Instituto Politécnico NacionalIrapuato, Mexico; ^3^Department of Biotechnology and Biochemistry, Cinvestav Unidad IrapuatoIrapuato, Mexico; ^4^Plant-Food Molecular Breeding Unit, Tecnologico de MonterreyMonterrey, Mexico

**Keywords:** maize (*Zea mays*), insect resistance, peroxidase, activity-directed proteomics, low-abundance proteins, plant proteomics

## Abstract

Plant peroxidases (PODs) are involved in diverse physiological processes, including defense against pathogens and insects. Contrary to their biological importance, only very few plant PODs have been proven on protein level, because their low abundance makes them difficult to detect in standard proteomics work-flows. A statistically significant positive correlation between POD activity and post-harvest insect resistance has been found for maize (*Zea mays*, p84C3) kernels. In combining activity-directed protein purification, genomic and proteomic tools we found that protein B6T173 (ZmPrx35) is responsible for the majority of the POD activity of the kernel. We successfully produced recombinant ZmPrx35 protein in *Escherichia coli* and demonstrate both, *in vitro* activity and the presence of a haem (heme) cofactor of the enzyme. Our findings support the screening for insect resistant maize variants and the construction of genetically optimized maize plants.

## Introduction

Post-harvest loss of maize due to insect pests is a serious problem and can reach up to 80% in tropical regions (Pingali and Pandey, 2001). E.g., the lowland tropics of Mexico suffer up to 100% kernel damage and 30% weight loss during half a year of storage (Bergvinson, 2001). Mainly small stockholders are affected, since they are not able to invest in suitable infrastructure and materials to protect their products. Maize kernels can be protected from insect damage either mechanically, by the use of metal silos (Tefera et al., 2011) and hermetic storage bags (García-Lara et al., 2013), or chemically, employing insecticides (Dales and Golob, 1997). However, both strategies increase the production costs for the farmer. In addition, the use of agrochemicals is discussed and is controversial due to environmental and health hazards (Pedlowski et al., 2012). Therefore, innate insect-resistance for maize kernels would be an attractive trait for plant breeding (Bergvinson and García-Lara, 2004).

Previous studies have shown a positive correlation between peroxidase (POD) activity and maize weevil (*Sitophilus zeamais*) resistance of maize kernels (García-Lara et al., 2007a; Winkler and García-Lara, 2010). In the UniProt^1^ database (Magrane and Consortium, 2011), more than 400 *Zea mays* proteins are tagged as PODs. Remarkably, experimental evidence on the protein level is reported for only three of them. All of those refer to the same study, describing guaiacol POD activities isolated from corn root plasma membranes (Mika and Lüthje, 2003). Even considering that more POD identifications might exist, which are not registered in the UniProt repository, a lack of biochemical knowledge about PODs is evident. PODs, classified as E.C.1.11.1.x, catalyze various oxidative reactions, employing peroxides (ROOH, mostly as H_2_O_2_) as electron acceptors (Fleischmann et al., 2004; Fawal et al., 2013). In plants, PODs participate in many physiological processes (for review see Hiraga et al., 2001), such as auxin metabolism (Lagrimini et al., 1997), lignination (Whetten et al., 1998), tolerance against osmotic stress (Amaya et al., 1999) and senescence (Abeles et al., 1988). Cell wall associated class III PODs are involved in the loosening and stiffening of cell walls during plant development. However, the detailed functions of individual PODs remain to be elucidated (Francoz et al., 2015).

Therefore, in a previous study we developed a proteomic work-flow, which permits the efficient screening for proteins with POD activity from 1D-SDS-PAGE gels (Winkler and García-Lara, 2010). However, in some cases a clear identification of the proteins responsible for the POD activity is hampered, since only partially separated protein fractions are studied.

With the 1D strategy, no classic POD was detected in extracts of the highly maize weevil resistant maize p84C3. Instead, an abundant protein of unknown function was identified: B4FFK9_MAIZE (UniProt accession code; Winkler and García-Lara, 2010). Consequently, we tested the POD activity of this protein after heterologous production in *Escherichia coli* (*E. coli*). Recombinant B4FFK9_MAIZE displayed neither POD activity nor the typical Soret peak of hemoproteins. This negative result indicated that the initial proteomic analysis was distorted by the low abundance of the active POD(s) relatively to other proteins in the kernel.

In order to achieve a reliable identification of the active POD in highly insect-resistant p84C3 maize kernels, we performed an activity-directed purification prior to the mass spectrometry based protein identification. Subsequently, we verified our results by amplification and cloning of the cDNA of interest, as well as recombinant production and biochemical studies of the putative POD.

## Materials and Methods

### Maize Genotypes

The open pollinated population p84 was developed at the International Maize and Wheat Improvement Center (CIMMYT) from twenty Caribbean accessions that possessed moderate resistance to the larger grain borer *Prostephanus truncatus* (Horn; García-Lara et al., 2004). For the proteomic analyses we chose the third selection cycle with incremented POD activity and insect resistance.

### 1D-GE, SDS-PAGE

Non-reducing SDS-PAGE and POD activity staining were carried out as described previously (Winkler and García-Lara, 2010). In short, maize seeds were milled using a Mixer Mill MM301 (Retsch, Hann, Germany) during 20 s at 30 Hz. Protein was extracted incubating 100 mg of tissue in 600 μL of 50 mM sodium phosphate buffer pH 6.8 and analyzed in SDS-PAGE 10%. The POD activity was detected after incubation with a solution of 20 mM guaiacol in 50 mM phosphate buffer pH 6.8 and 3% H_2_O_2_ for 30 min. As a second staining step, the protein in the gel was fixed in 40% (*v/v*) ethanol and 10% (*v/v*) acetic acid for 1 h and stained with Brilliant Blue R250 0.1% solution (Sigma-Aldrich, St. Louis, MO, USA).

### Two-Dimensional (2D) Gel Electrophoresis

Two-dimensional gel electrophoresis was performed according to the method of Bjellqvist et al. (1982), and carried out as reported previously by our group (Mata-Gómez et al., 2012). However, the conditions were adjusted in order to prevent the loss of POD activity. In particular, boiling and reducing agents were eliminated from the procedure. Immobiline^TM^ dry strips of 13 cm length (GE Healthcare, Uppsala, Sweden) were rehydrated 12 h at 20°C with non-reductive isoelectric focusing buffer (50 mM Tris-HCl buffer pH 6.8, 0.5% ampholite, 2% CHAPS and 0.0001% bromophenol blue), containing 150 μg of protein. IEF was conducted with an Ettan IPGphor 3 (GE Healthcare, Uppsala, Sweden). Focusing of pH 6–11 strips was carried out as follows: 150 V for 1 h, 300 V for 1 h, 600 V for 1 h followed by 8,000 V in gradient for 0.5 h and finally 5,000 V until reaching 26,000 Vh. For the pH 4–7 strips, the focusing was performed 250 V for 1 h, 500 V for 0.5 h, followed by 1,000 V for 0.5 h and finally 8,000 V to reach 12,000 Vh. After focusing, the gels were equilibrated twice for 15 min in a solution containing 6 M urea, 30% *w/v* glycerol, 2% w/v SDS and 50 mM Tris-HCl buffer, pH 8.8. For the second dimension, the proteins were separated on 12% SDS polyacrylamide gels. The POD activity was observed incubating the gels with a solution of 20 mM guaiacol in 50 mM phosphate buffer pH 6.8 and 3% H_2_O_2_ for 30 min. The complete profile of protein spots was visualized using 0.1% Brilliant blue R250 (Sigma-Aldrich, USA).

### Densitometric Analysis

For the quantification of the POD activity on 2D gels, an image analysis was performed by using the plugin Yawi-2D of the free Software ImageJ 1.47a. For further spot intensity determination, performed by the comparison of the integrals of color density of each spot, the original image was converted to an eight bit-grayscale image with 300 dpi resolution.

### In-Gel Digestion of Protein Bands

For in-gel digestion of protein bands, the Shevchenko protocol (Shevchenko et al., 1996, 2006) was slightly modified, as described previously (Winkler and García-Lara, 2010). After the SDS-PAGE, the POD active spots were sliced from the gel and chopped into cubes with about 1 mm of edge length. The cubes were transferred to vials and washed with a 1:1 (*v/v)* solution of 125 mM ammonium bicarbonate and acetonitrile (ACN) until complete discolouration. The reduction and alkylation steps were performed by incubation with 10 mM DTT and 55 mM IAA. The gel pieces were rinsed with ACN, then the shrunken gel pieces were dried in a vacuum centrifuge. For the protein digestion, the dry gel pieces were re-hydrated in a 50 mM ammonium bicarbonate solution containing 10 ng/L trypsin (PROMEGA, Madison, WI, USA) and incubated overnight at 37°C. After tryptic digestion, the peptides could be extracted by shaking for 15 min with a 1:2 (*v/v*) solution of 5 % formic acid/ACN at 37°C. The supernatant was transferred to a new tube and dried in a vacuum centrifuge. Prior to LC-MS/MS analysis, the peptides were dissolved in 20 μL of 0.1% (*v/v*) formic acid.

### Nanoflow LC-MS/MS

All experiments were performed on a nanoAcquity nanoflow liquid chromatography (LC) system (Waters, Milford, MA, USA), coupled to a linear ion trap LTQ Velos mass spectrometer (Thermo Fisher Scientific, Bremen, Germany), equipped with a nano electrospray ion source. Solvent A consisted of 0.1% formic acid and solvent B of 100% ACN with 0.1% formic acid. Three micro liter of tryptically digested proteins were bound to a pre-column (Symmetry^®^ C18, 5 μm, 180 μm × 20 mm, Waters). Subsequently, the flow was then switched to a 10 cm capillary UPLC column (100 μm ID BEH-C18 1.7 μm particle size). The column temperature was controlled at 35°C. The peptides were separated by a 60 min gradient method at a flow rate of 400 nL/min. The gradient was programmed as follows: 3–50 % solvent B (over 30 min), 50–85% B (over 1 min), 85% B (for 7 min) and 3% B (over 22 min). The peptides were eluted into the mass spectrometer nano electrospray source through a standard coated silica tip (NewObjective, Woburn, MA, USA). The mass spectrometer was operated in data-dependent acquisition mode in order to automatically alternate between full scan (400–2000 *m/z*) and subsequent CID and PQD MS/MS scans in the linear ion trap. CID was performed using helium as collision gas at a normalized collision energy of 40% and 10 ms activation time. Data acquisition was controlled by Xcalibur 2.0.7 software (Thermo Fisher Scientific).

### Recombinant B4FFK9 Production

For the recombinant production of B4FFK9 protein, a synthetic and codon- optimized version of the gene was designed (GenScript, Piscataway, NJ, USA). This gene was cloned into the pGEX-6P-1 vector (GE Healthcare, Uppsala, Sweden), between the restriction sites BamHI and EcoRI. The protein production was performed in the *E. coli* BL21 (DE3) Rosetta gami strain, using 0.5 mM IPTG as inductor and 1 mM 5-aminolevulinic acid (ALA, SIGMA-Aldrich, St. Louis, MO, USA) as cofactor supply. The induction was performed at 16°C during 16 h. After the incubation, the bacterial pellet was recovered and re-suspended in 25 mM Tris-HCl, pH 7.0, buffer with 100 mM NaCl. The protein was purified with a standard GST purification procedure, using a Glutathione Sepharose column (16 × 25 mm.; GE Healthcare, Uppsala, Sweden), and according to the column manual. Imidazole was removed from the protein fractions by dialysis. The purified protein was cleaved from the GST–tag using a recombinant PreScission Protease, and tested for POD activity. POD activity was tested by incubating 20 μL of protein solution in 230 μL of reaction buffer (50 mM sodium phosphate pH 6.8, 20 mM guaiacol, 0.3% H_2_O_2_).

### Partial Purification of Native Peroxidases from Maize Seeds

Native PODs were partially purified in a three-step strategy: 50 g of milled maize seed tissue were homogenized with 250 mL of 25 mM Tris-HCl, pH 7.0, then incubated at 4°C during 1 h and subsequently centrifuged at 27,150 *g* for 30 min. The supernatant was collected, filtered and then loaded on a Macro-Prep High S Support column (1.5 × 14.1 cm; Bio-Rad, Hercules, CA, USA). Protein was eluted with a linear gradient from 0 to 1 M NaCl. A POD activity test was performed for all collected fractions as described above. The fractions with POD activity were then affinity-separated using a Concanavalin A column (0.8 × 4 cm.; SIGMA, USA) and tested again for POD activity. Active fractions were dialysed against 25 mM Tris pH 7.0 and centrifuged at 20,400 *g* for 5 min. The supernatant was collected and purified using a 5 mL “Macro-Prep High Q Support” column (Bio-Rad, Hercules, CA, USA). The flow-through was collected and concentrated using ultrafiltration membranes (10 kDa MWCO, Millipore, USA). The band with POD activity was separated by SDS-PAGE and prepared for nanoESI-LC-MS/MS.

### Maize RNA Extraction and cDNA Preparation

RNA was extracted from p84C3 maize seeds according to the protocol of Wang et al. (2012). cDNA was prepared using the SuperScript III Reverse Transcriptase (Invitrogen, Carlsbad, CA, USA) and a Poly-dT primer, following the manufacturer indications for GC-rich genes. The reaction mixture was heated at 65°C for 5 min. The elongation was carried out with a temperature of 50°C.

### ZmPrx35 Gene Constructs

For the recombinant production of B6T173 (ZmPrx35) protein, the respective gene was amplified from p84C3 maize cDNA using an N-terminal primer including a NdeI restriction site at the start codon: 5′-GACGACGACATATGAGCTCGACGTGGCTGGC-3′ and a C-terminal primer including a BamHI restriction site downstream of the stop codon: 5′-TCGTCGTCGGATCCCTAGTAGTGTGGGTTGACGA-3′. The amplification was performed considering the high GC content of the gene, using the Kapa HiFi Polymerase (Kapa Biosystems, Wilmington, MA, USA). For PCR, the following conditions were used: after denaturation at 95°C for 5 min, 35 cycles were carried out with: 20 s of denaturation at 98°C, 15 s of annealing at 57.5°C and 1 min of extension at 72°C. Finally, a denaturation temperature of 72°C was held for 10 min. The amplicon was cloned into different vectors for testing protein production: pET19b, pET28b, and pMALc5x, using the restriction sites NdeI and BamHI.

### Recombinant B6T173 (ZmPrx35) Production

Protein production for the constructs cloned into the pET19b and the pMALc5x vectors was performed using the *E. coli* BL21 (DE3) Rosetta gami strain. For the construct cloned into the pET28b vector, the *E. coli* BL21 (DE3) Rosetta II start was used. In both cases, the cultures were grown to an OD_600_ of 0.8 before inducing the gene expression with 0.5 mM IPTG. 1 mM ALA, 1 mM FeSO_4_ and 1 mM CaCl_2_ were supplemented as cofactors. The induction was carried out at 16°C for 16 h. The bacterial pellet was recovered and re-suspended in 25 mM Tris-HCl pH 7.0, 2 M NaCl buffer. The protein was purified in four steps: (1) A standard “His-Tag” affinity chromatography procedure, using a His-Trap FF column (16 × 25 mm; GE Healthcare, Uppsala, Sweden); (2) and (3) two ionic-exchange purification steps, using columns Q (0.8 × 4 cm, High Q support, Bio Rad, USA) and S (0.8 × 4 cm, High S support, Bio Rad, Saint Louis, MO, USA), and (4) a size-exclusion chromatography on a Superdex 75 Column (10 × 300 mm; GE Healthcare, Uppsala, Sweden). The purified fractions were concentrated by ultrafiltration.

### Optimized B6T173 (ZmPrx35) Production

For increasing yield and purity of the recombinant ZmPrx35 protein, an optimized version of the gene was designed and divided into two gBlocks (Integrated DNA Technologies, USA). The gBlocks were cloned independently into the pJET1.2 vector (Thermo Scientific, USA), and then fused using a previously described protocol (Heckman and Pease, 2007). The assembled gene was cloned into the vectors pET32a and pET28b (Novagen), between the restriction sites EcoRI and Xho I. The protein production was performed in the *E. coli* BL21 (DE3) Rosetta gami strain, using 1 mM IPTG as inductor and supplementing the culture media with 1 mM ALA and 1 mM FeSO_4_ as cofactors. The induction was carried out at 16°C for 16 h. After the incubation, the bacterial pellet was recovered and re-suspended in a 25 mM Tris-HCl pH 7.0, 150 mM NaCl buffer. For the purification assay, the standard “His-Tag” purification procedure was tested, using a His-Trap FF column (16 × 25 mm, GE Healthcare, Uppsala, Sweden).

### Identification of the Haem Cofactor

The identification of the haem group was achieved by two different strategies. The first strategy relied on a spectro-photometrical scanning from 250 to 800 nm, in order to detect the Soret peak of absorbance, which is expected between 400 and 500 nm (Dalton et al., 1996; Shannon et al., 1966; Ray et al., 2012).

The second strategy was a luminol-based test for iron detection. This assay was performed as described by Högbom et al. (2005). Two different conditions have been tested: the protein in native state, and the unfolded (denatured) protein. For the protein in native state, 1 μg of protein were loaded on a black, half area, 96-well plate (Corning, NY, USA), followed by the addition of 40 μL of 1 M Tris-HCl pH 7.0 and 100 μL of the reaction buffer (11 mM luminol, 500 mM Na_2_CO3, 230 mM H_2_O_2_). For the unfolded protein, 40 μL of 8 M urea were loaded to the plate, followed for the addition of the same amounts of protein and reaction buffer described above. All the buffer solutions were treated with Chelex 100 (Sigma, St. Louis, MO, USA). The luminescence was detected by exposing a CL-Xposure Film (Thermo Scientific, Rockford, IL, USA) to the wells of the plate with the reaction mixture.

### Protein Identification and Hit Validation

Employing a target-decoy strategy (Elias and Gygi, 2007), concatenated databases were generated. For the native maize protein identification, the decoy database contained all *Z. mays* entries of the NCBI protein database^2^ (download 13/04/24). For recombinantly produced protein, the decoy database included all *E. coli* BL21(DE3) entries (^2^download 15/04/27) and the B6T173 entry of *Z. mays*. Generation of databases was performed by using the software FastaTools 0.9 (David Ovelleiro, CSIC-UAB). Raw spectra were converted to *^∗^.mzXML* and *^∗^.mgf* files using the ProteoWizard toolkit version 3.0.3364 (Chambers et al., 2012).

Data were analyzed by using two different proteomic platforms. In the first strategy, the data were uploaded to a local LabKey 12.3 server (Rauch et al., 2006) and subsequently analyzed with a pipeline employing the bundled versions of X!Tandem (Craig and Beavis, 2004), PeptideProphet (Keller et al., 2002) and ProteinProphet (Nesvizhskii et al., 2003). For the ProteinProphet, a minimum protein probability of 0.95 was set.

In the second strategy, we transformed the Thermo raw data with to ProteoWizard toolkit to *^∗^.mgf* and performed an analysis with the PeptideShaker suite v0.38.1 (Barsnes et al., 2011). A PeptideShaker compatible target-decoy database was built from the UniProt *Z. mays* protein sequences (‘un-reviewed’) and submitted to the bundled SearchGUI tool (Vaudel et al., 2011). As search engines we employed in this analysis both possible options, OMSSA version 2.1.9 win32 (Geer et al., 2004) and X!Tandem version 13.2.1.1 (Craig and Beavis, 2004).

We re-processed the mass spectrometry data with the Trans-Proteomic Pipeline 4.8.0^3^ (Deutsch et al., 2015) on MASSyPup (Winkler, 2014). Raw files and results were deposited to the ProteomeXchange Consortium^4^ (Vizcaíno et al., 2014) via the PRIDE partner repository^5^. The data may be accessed with the login reviewer64284@ebi.ac.uk and password Td4Dkr5d, using the dataset identifier PXD002166 (after publication of the article, the data will be public).

### BLAST Searches

BLAST searches were performed using BLASTP (Altschul et al., 1997) on the UniProt webpage^6^, searching the UniProtKB (Magrane and Consortium, 2011) database with the standard parameters.

### Maize eFP Browser

The expression pattern of the identified protein was investigated with the Maize eFP Browser^7^ (Winter et al., 2007; Sekhon et al., 2011).

## Results and Discussion

### Proteins with Peroxidase Activity in 2D Gel Analysis

**FIGURE 1:**
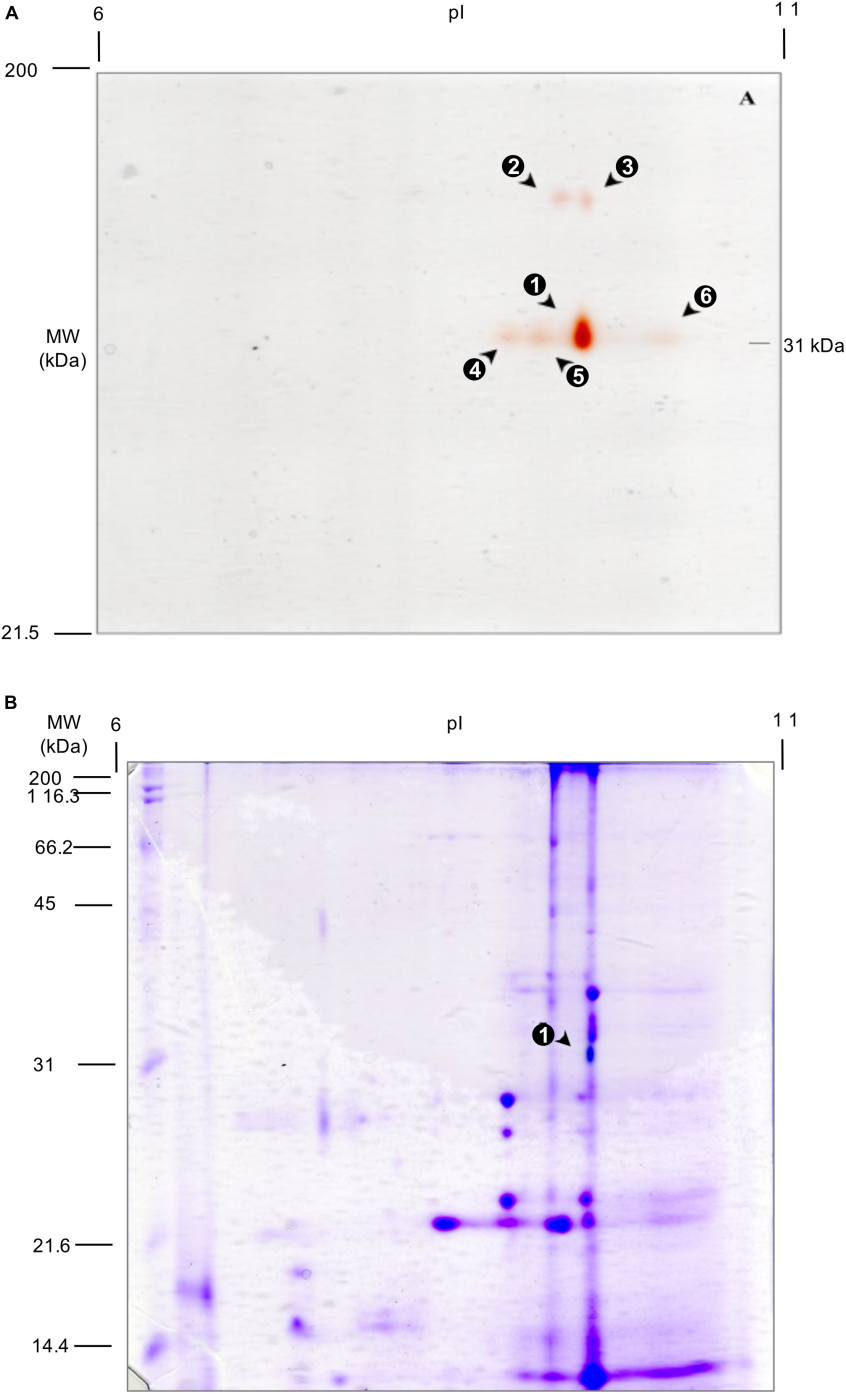
**2D Analysis of peroxidase (POD) activity from p84C3 maize kernels. (A)** Guaiacol-H_2_O_2_ staining. **(B)** Sequential staining (guaiacol-H_2_O_2_ + Coomassie R-250). Arrows point to the spots with POD activity.

Separation of proteins from p84C3 maize kernel extracts with 2D electrophoresis and subsequent staining for POD activity revealed six defined active spots (**Figure 1A**). Densitometric analysis suggests that spot 1 accounts for about 80% of the total activity (see **Table 1**). This is astonishing because more than 400 PODs are suspected for *Z. mays* according to the UniProt^8^ database. Spot 1 exhibits an apparent MW of 31 kDa and a pI of 9.5. Spots 4–6 display the same apparent molecular weight on the gel and thus might be isoforms of the protein represented by spot 1. Spots 2 and 3 displayed an apparent MW of 45 kDa and pI of 9.3 and 9.5, respectively. After subsequent Coomassie staining of the gel, only one protein spot was still visible (**Figure 1B**). This spot corresponds to the Spot 1 of the guaiacol-H_2_O_2_ staining (**Figure 1A**). The protein of this spot was sliced from the gel and subjected to nanoLC-MS/MS analysis.

**Table 1:** Densitometric evaluation of peroxidase activity in 2D gel analysis.

Spot	% of total activity
1	80.6
2	3.12
3	3.78
4	3.34
5	6.89
6	2.24

Six proteins with more than two identified peptides were found in spot 1 by using PeptideShaker (see **Table 2**). The identification of various proteins in a presumably separated spot is not uncommon (Gupta et al., 2015), considering the high sensitivity of nanoLC-MS/MS and presence of highly abundant proteins, which contribute to the background. Ten validated peptides belong to Lactoylglutathione lyases. Further, late embryogenesis abundant protein D-34 was identified, as well as two isoforms of embryonic protein DC-8. None of those proteins can explain the POD activity. Two identified proteins are not annotated in the UniProt database and were submitted to BLAST searches. The UniProt protein sequences K7VEA3 and K7VM99 are isoforms and show a 66–68% identity with Uniprot entry K4F957, a late embryogenesis abundant protein from *Oryza sativa* subsp. *japonica* (Rice). B4FFK9 displays 81 % identity with Uniprot entry Q94J20, a lipoprotein-like *Oryza sativa* subsp. *japonica* (Rice) and 63 % identity with the secreted *Z. mays* (Maize) proteins B6UI56 and K7V532. B4FFK9 corresponds to the accessions GRMZM2G043521_T01 and GRMZM2G043521_P01 in the maize sequence database^9^. Analysis of the expression pattern in the eFP Browser reveals a high abundance of the transcript GRMZM2G043521_T01 in the embryonic tissue during the last phase of embryogenesis, but it is absent in any other maize tissue or in other phases of plant development (Supplementary Figure S2), which suggests a specialized role for the maize kernel.

**Table 2:** NanoESI-LC-MS/MS based identification of proteins from 2D gel, spot 1, after POD activity staining (Data analysis with LabKey).

Hit	Protein group	Inference class	Description	Sequence coverage [%]	#Validated peptides	#Validated spectra	Theoretical MW [kDa]
1	B6TPH0, C0PK05	Unrelated proteins	Lactoylglutathione lyase	21.3	10	22	35.1
2	B6UH67	Single protein	Late embryogenesis abundant protein D-34	41.7	7	11	27.2
3	B6SGN7	Single protein	Embryonic protein DC-8	16.1	6	12	32.3
4	B4FFK9	Single protein	Uncharacterized protein/Lipoprotein^1^/ Secreted protein^1^	19.0	5	6	27.7
5	B6TK66	Single protein	Embryonic protein DC-8	10.3	2	2	33.6
6	K7VEA3, K7VM99	Isoforms	Uncharacterized protein/ Late embryogenesis abundant protein^1^	12.5	2	2	28.4

*^1^Evaluated by BLASTP*.

### *In Vitro* Production of B4FFK9 in *E. coli*

From the 2D gel analysis and subsequent protein identification, as well as considering the expression profile, B4FFK9 was the most likely POD protein candidate. Additionally, local sequence similarity with POD motifs could indicate a novel POD type (Winkler and García-Lara, 2010). Thus, we tried a recombinant production of the protein in *E. coli*, as described in the methods part. The features of the purification buffers, near to neutral conditions, were designed according to the results of the 2D analysis (**Figure 1**), where the most active POD exhibited a pI near 9.5. Strong protein production was observable (Supplementary Figure S1B), but no POD activity could be detected (Supplementary Figure S1A). UV-VIS spectra did not show the expected Soret-band features (data not shown), indicating the absence of a haem cofactor. Suspecting incomplete cofactor loading, we supplemented the protein production with 5-ALA. But in none of the experiments we detected active protein.

Currently, plant PODs for industrial or laboratory use are either extracted from biological material or produced *in vitro* in cell cultures (González-Rábade et al., 2012). In some cases, recombinant versions of PODs are susceptible to bacterial oxidative stress, by a peroxide-mediated inactivation process (Arnao et al., 1990), resulting in a recombinant non-active version of the enzyme. Since we could not verify the POD activity of B4FFK9, we decided to track down other POD candidates by an activity-directed purification strategy.

### Partial Purification of Peroxidases and 1D-GE/ NanoLC-MSMS Identification

**FIGURE 2:**
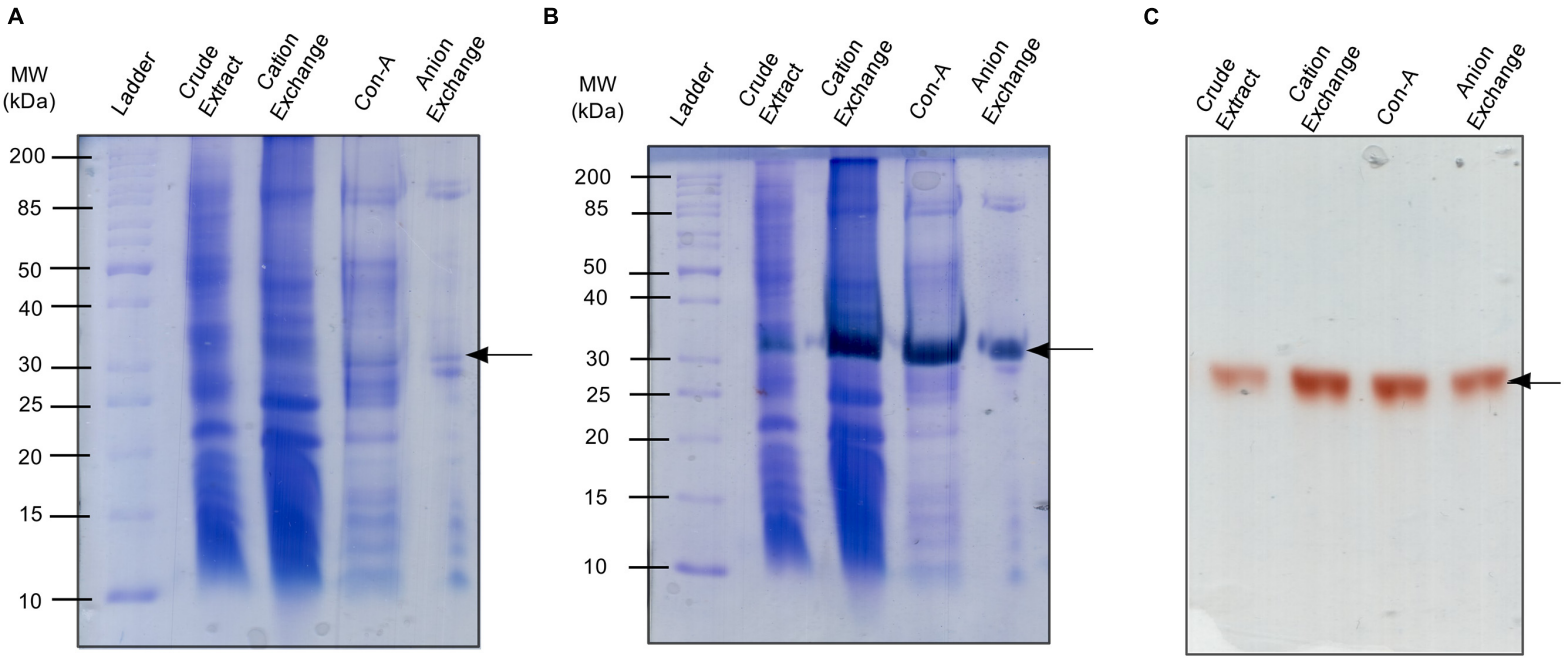
**Purification of protein with POD activity from p84C3 maize kernels. (A)** Coomassie R-250 staining of the POD fractions of the different purification steps. **(B)** Sequential staining of POD fractions (guaiacol-H_2_O_2_ + Coomassie R-250). **(C)** Guaiacol staining of the same fractions. In all cases, 20 μL of protein of the indicated fraction was loaded on each lane. Arrows indicate bands with POD activity.

Activity-directed partial purification of the most active POD from p84C3 seeds revealed a rarefied, yet highly active, protein (**Figure 2**). The apparent MW of the semi-purified enzyme is congruent with the spot location of the POD active protein in the 2D analysis.

In subsequent 1D-GE/nanoLC-MSMS analyses we identified various protein candidates (see **Table 3**). The proteins B6T173_MAIZE and K7TID5_MAIZE are predicted as PODs in the UniProtKB database, although no experimental evidence for their existence on protein level was reported (Magrane and Consortium, 2011). The location of spot is congruent with the molecular weight of the protein, but the apparent pI of the purified POD is more basic than the predicted value for B6T173. We contribute this pI shift to the post-translational modification with the Fe-containing cofactor and glycosylation.

**Table 3:** Identification of proteins from semi-purified fraction in 1D gel band with peroxidase activity, using nanoESI-LC-MS/MS (Data analysis with PeptideShaker).

Hit	Protein Group	Inference Class	Description	Sequence Coverage [%]	#Validated Peptides	#Validated Spectra	Theoretical MW [kDa]
1	B4G1C2	Single protein	Uncharacterized protein	66.99	24	473	34.21
2	K7TID5	Single protein	Peroxidase^1^	42.77	13	25	36.77
3	B6T173	Single protein	Peroxidase^1^	53.98	13	26	36.8
4	B4G1D7	Single protein	Uncharacterized protein	29.13	7	18	38.8
5	B8QV73	Single protein	Chtinase	52.3	8	38	29.27
6	B6SGT3	Single protein	Xylanase Inhibitor protein^1^	35.71	11	82	33.1
7	B8QV49	Single protein	Chtinase	42.86	6	26	28.8

*^1^Evaluated by BLASTP*.

Apart from these highly probable POD candidates, we found two chitinases (B8QV73_MAIZE and B8QV49_MAIZE) and a xylanase inhibitor (B6SGT3_MAIZE) in the fraction. Two uncharacterized proteins (B4G1C2_MAIZE and B4G1D7_MAIZE) we excluded as possible PODs after a sequence analysis and domain predictions.

**FIGURE 3:**
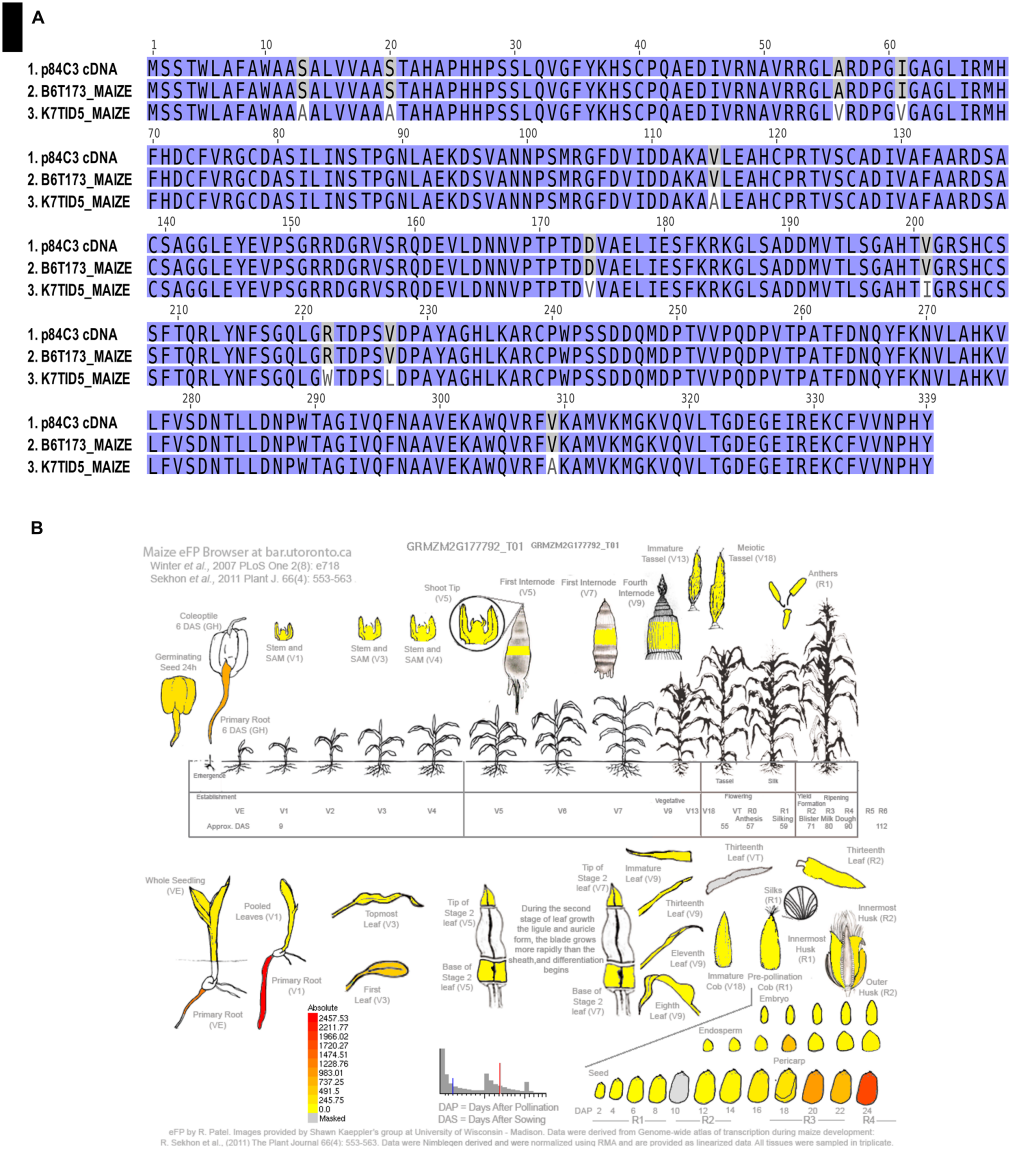
**Identification and expression patterns of the POD isolated from cDNA. (A)** Alignment showing the similarity of the gene product isolated from the cDNA of p84C3 maize kernels with the two putative candidates: B6T173_MAIZE and K7TID5_MAIZE. **(B)** Expression patterns of the accession GRMZM2G177792_T01 accession in B73 maize (corresponding to B6T173_MAIZE UniProt accession) using the Maize eFP Browser (http://www.bar.utoronto.ca/).

The peptidic sequences of the POD candidates B6T173_MAIZE and K7TID5_MAIZE only differ in ten amino acid residues, as shown in **Figure 3A**, which could suggest the presence of isoforms of the enzyme. B6T173_MAIZE corresponds to the accession GRMZM2G177792_T01 in the maize sequence database^10^ and to the PeroxiBase entry ZmPrx35, which has been predicted as a Class III POD (Fawal et al., 2013; Wang et al., 2015). For K7TID5_MAIZE we found no further information in publicly accessible databases or literature.

### Identification of B6T173 from *Z. mays* p83C3 cDNA

Since both possible POD candidates B6T173_MAIZE and K7TID5_MAIZE share an identical N- and C-terminal region in the amino acid sequence (**Figure 3A**), we designed primers to amplify the actual gene from *Z. mays* p84C3 cDNA. Sequencing of the PCR product revealed 100% amino acid sequence homology with the B6T173 protein.

### *In Vitro* Production of B6T173 (ZmPrx35) in *E. coli* and Confirmation of Activity, Identity, and Haem Cofactor

With the expression of the cloned p84C3 cDNA B6T173 amplification product, we were able to obtain recombinant and active protein, by using the pET19b, pET28b, and pMALc5x vectors (Supplementary Figure S4). The construct using the pET19b vector exhibited more activity than the other constructs (Supplementary Figure S4A). Expression of codon-optimized B6T173, cloned into the pET32a vector resulted in abundant but inactive protein product. The loss of function in the pET32a construct could be due to misfolding or an inhibition of the POD by the co-expressed thioredoxin.

**FIGURE 4:**
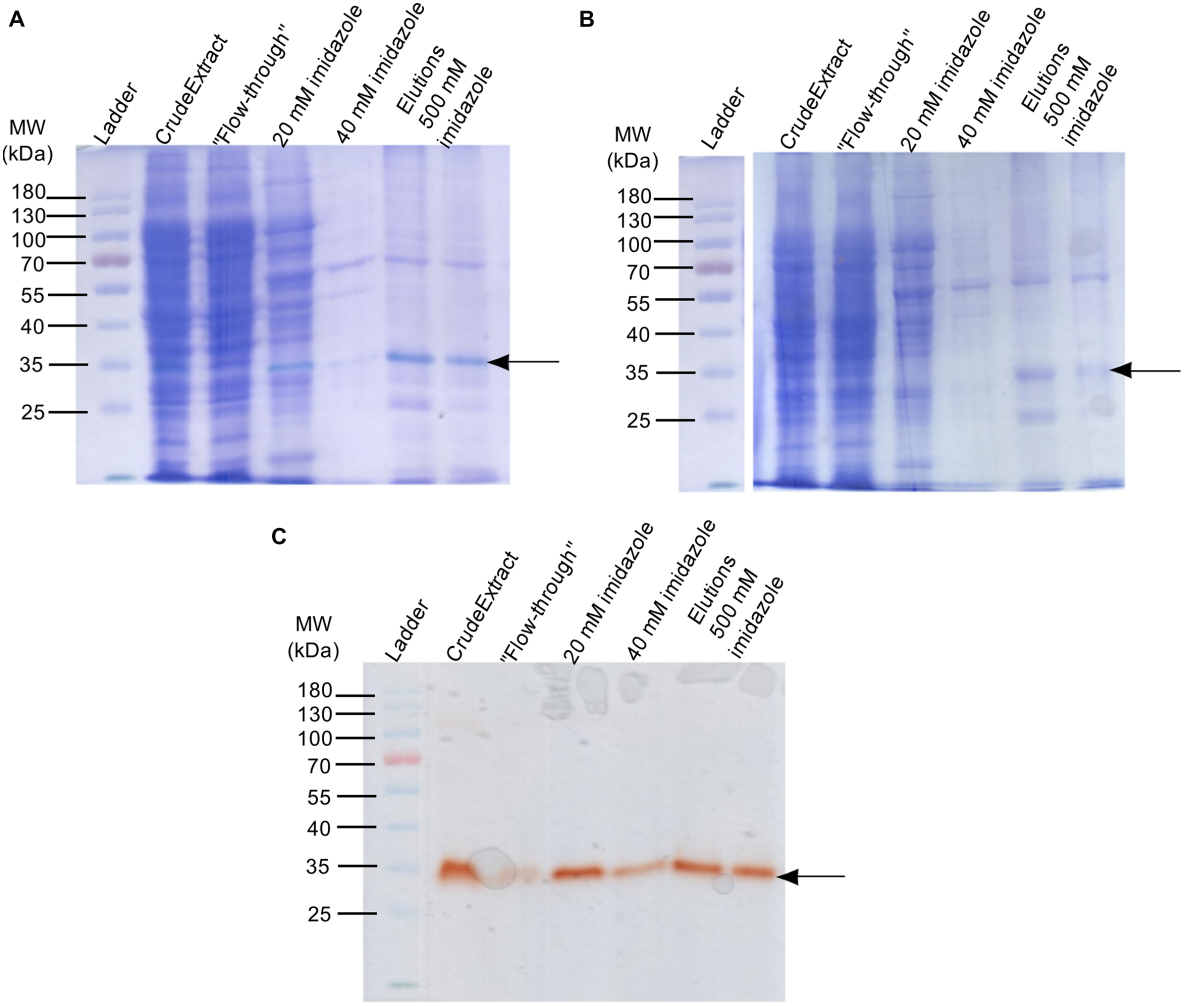
**Production and purification of the recombinant B6T173 (ZmPrx35) protein in *E. coli* (using the vector pET19b). (A)** Sequential staining (guaiacol- H_2_O_2_ + Coomassie R-250) of the POD fractions obtained by IMAC affinity purification. **(B)** Coomassie R-250 staining of the POD active fractions. **(C)** Guaiacol-H_2_O_2_ staining of the same POD fractions. In all cases, 20 μL of protein solution were loaded. Arrows indicate bands with POD activity.

The recombinant active protein obtained from the pET19b construct expression has an approximate MW of 34 kDa, which is congruent with the B6T173 amino acid sequence (**Figure 4**). NanoLC-MS/MS based identification verified the presence of B6T173_MAIZE (ZmPrx35) in the band with POD activity from the 1D gel with semi-purified fractions of recombinant *E. coli* production (see **Table 4**). Since no POD activity is detected in non-induced cultures (Supplementary Figure S4A), the results provide strong evidence for the heterologous production of active ZmPrx35 POD. The presence of activity after production in a bacterial host suggests that B6T173_MAIZE activity is independent from glycosylation. Similar findings were reported for the soybean cytosolic ascorbate POD (Dalton et al., 1996) and catalase (Ray et al., 2012).

**Table 4:** Verification of identity of recombinant B6T173 (ZmPrx35) by nanoESI-LC-MS/MS (Data analysis with PeptideShaker).

Hit	Protein group	Inference class	Description	Sequence coverage [%]	#Validated peptides	#Validated spectra	Theoretical MW [kDa]
1	B6T173	Single protein	Peroxidase (*Zea mays*)	19.17	6	8	36.8
2	C6EEI6^1^	Single protein	Predicted DNA (exogenous) processing protein	12.14	3	5	31.92
3	C6EL61^1^	Single protein	Phosphoenolpyruvate-protein phosphotransferase	1.91	1	1	63.49
4	C6EK20^1^	Single protein	Rnase III	5.31	1	2	25.53
5	C6EG51^1^	Single protein	D-ribose transporter subunit	10.47	1	2	30.92
6	C6EB56^1^	Single protein	6-phosphogluconate dehydrogenase, decarboxylating	2.49	1	1	51.46
7	C6EKH4^1^	Single protein	Inositol monophosphatase	4.87	1	1	29.15

*^1^Escherichia coli host protein*.

**FIGURE 5:**
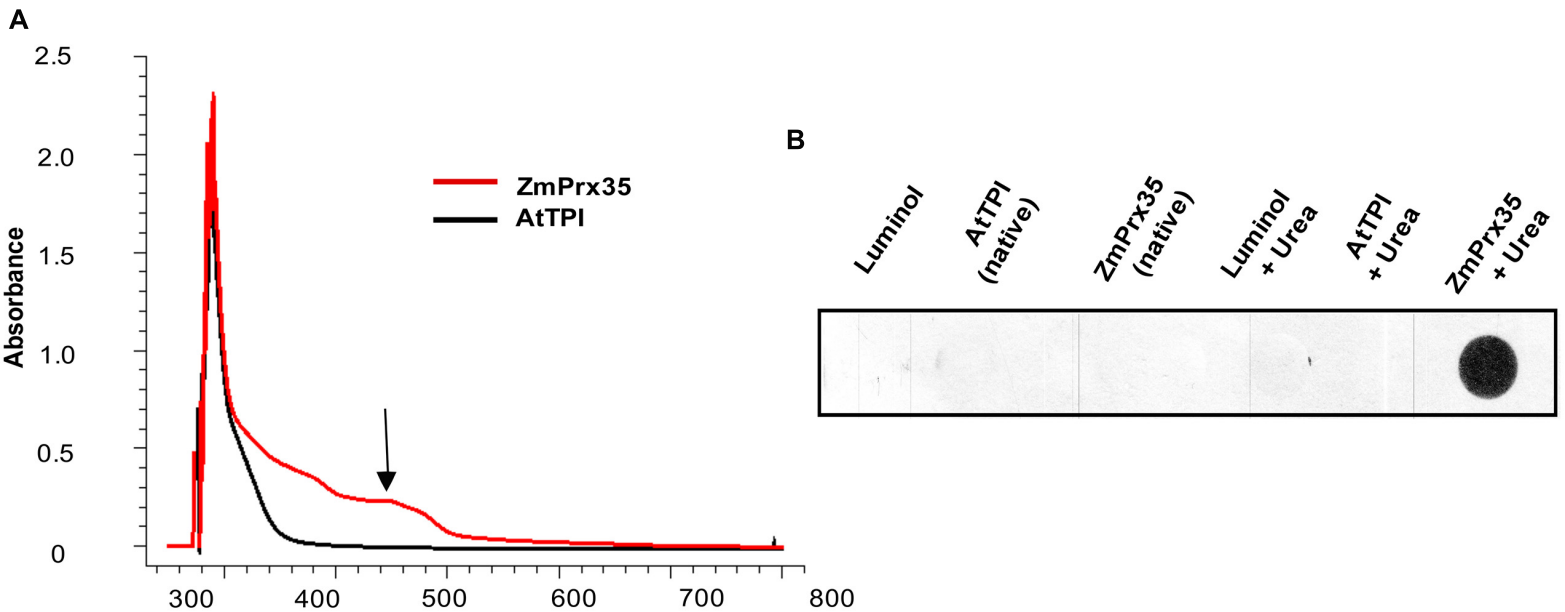
**B6T173 (ZmPrx35) iron cofactor detection. (A)** Spectra of absorbance (200-800 nm) of ZmPrx35 purified fractions, and of a negative control (At CytoTPI). The arrow shows the peak of absorbance at 450 nm (Soret band). **(B)** Quantitative luminol assay of ZmPrx35 and AtCyto TPI (as a negative control) under native and unfolded conditions.

UV-VIS spectra of recombinant ZmPrx35 showed increased absorbance with a maximum at about 450 nm (**Figure 5A**), which corresponds to a Soret-band feature. Thus, the spectroscopic data indicate the presence a haem group, which is a main structural characteristic of PODs (Dalton et al., 1996; Shannon et al., 1966) and related haem-dependent redox proteins such as catalase (Ray et al., 2012).

Surprisingly, no iron – the central bio-metal of the haem cofactor - was detected in ZmPrx35 protein under native conditions, using the luminol assay (**Figure 5B**). However, after denaturing the protein with 8 M urea, the test was positive (**Figure 5B**), confirming the completeness of the POD haem cofactor.

We created a structural model of ZmPrx35 using the I-TASSER server (Supplementary Figure S3) (Yang et al., 2014). Most of the twenty-six residues predicted coordinate the haem group point toward the inside of the protein, and only a defined small channel allows entry to the active center harboring the haem group. The model is similar to other known structures of PODs (Østergaard et al., 2000; Watanabe et al., 2010). Thus, the experimental and the structural modeling results are congruent and indicate a hidden iron cofactor of the enzyme.

### Expression Profile Analysis

The transcript of GRMZM2G177792_T01 (corresponding to B6T173 protein) demonstrates a high abundance of the transcript in the last stages of the seed development, as well as in the primary root during the first stages of the development of the seedling (**Figure 3B**). The expression profile suggests a specialized function of the POD during seed dormancy and early plant development. Searching the EnsemblePlant database^11^, 67 orthologs and 45 paralogs of B6T173 can be identified in various cereals. In *Sorghum bicolor*, the ortholog gene Sb09g002830 displays 82% identity, the corresponding gene Si025196m.g in *Setaria italica* 81% identity. In none of the genes, the biological role has been confirmed, and only some of them have been predicted as possible PODs.

### Function of B6T173 (ZmPrx35) in Insect-Resistant Maize Kernels

To date there are few reports about PODs in maize. POD 1 and POD 70 (from corn roots) have been correlated with removal of H_2_O_2_, oxidation of toxic reductants, biosynthesis and degradation of lignin, suberization, auxin catabolism, response to environmental stresses such as wounding, pathogen attack and oxidative stress. These functions might depend on different isozymes/isoforms in disctinct plant tissues (Hiraga et al., 2001).

Seed PODs have been associated with various functions. Some of them are involved in the germination process, such as the barley POD isozymes (Laugesen et al., 2007) and the rice OsAPX1 (Kim et al., 2015).

A current study suggests that cross-linking of cell-wall polymers through ester-linked diferulates has a key role in plant resistance to corn borers, which is mainly due to kernel toughness rather than the indigestibility of the cell wall compounds (Barros-Rios et al., 2015). The main two enzyme classes of enzymes which are involved in this polymerization mechanism are polyphenol oxidases and PODs (García-Lara et al., 2004; Lattanzio et al., 2006; Barros-Rios et al., 2015). PODs catalyze the oxidative coupling of feruloyl polysaccharides and thus increase the firmness of the cell wall, especially in the presence of reactive oxygen species (ROS; Fry et al., 2000). This function has been suggested for other seed PODs, as the soybean anionic POD SP4.1 (Gillikin and Graham, 1991).

Thus, the observed positive correlation of endosperm POD activity with maize weevil resistance for maize populations (García-Lara et al., 2007b) could be attributed to a mechanical protection of the seed, which in turn is mediated by oxidative cross-linking reactions, catalyzed by PODs. Surprisingly, a single POD, B6T173 (ZmPrx35), seems to be responsible for the high insect resistance of p84C3 maize seeds.

## Conclusion

We identified and characterized the class III POD B6T173 (ZmPrx35), which accounts for about 80% of the POD activity in maize (*Z. mays* p84C3) kernels. The positive correlation between POD activity and post-harvest insect resistance suggests the use of ZmPrx35 as biomarker and for genetic engineering of maize.

Considering possible mechanisms of resistance and enzyme functions, ZmPrx35 is likely to be involved in the cell-wall strengthening by oxidative coupling of feruloyl polysaccharides. The expression pattern of the respective gene transcript indicates an additional role in the seedling development.

Despite their physiological relevance, only few plant PODs have been studied on protein level up to now. Therefore, to account for the low abundance of these enzymes we recommend an activity-directed proteomics strategy.

## Conflict of Interest Statement

The authors declare that the research was conducted in the absence of any commercial or financial relationships that could be construed as a potential conflict of interest.

## Footnotes

1 http://www.uniprot.org

2 http://www.ncbi.nlm.nih.gov/protein/

3 http://sourceforge.net/p/sashimi/wiki/Home/

4 http://www.proteomexchange.org/

5 http://www.ebi.ac.uk/pride/archive/.

6 http://www.uniprot.org/blast/

7 http://www.bar.utoronto.ca/

8 http://www.uniprot.org

9 http://www.maizesequence.org.

10 http://www.maizesequence.org

11 http://plants.ensembl.org/
