# Supplementary material for: Identification of B6T173 (ZmPrx35) as the prevailing peroxidase in highly insect-resistant maize (Zea mays, p84C3) kernels by activity-directed purification
Source: Front Plant Sci. 2015 Aug 31;6:670. doi: 10.3389/fpls.2015.00670 (PMC4553411; doi:10.3389/fpls.2015.00670)
Supplement: Supplementary file 1 [file Presentation_1.PDF]

SUPPLEMENTAL FIGURES

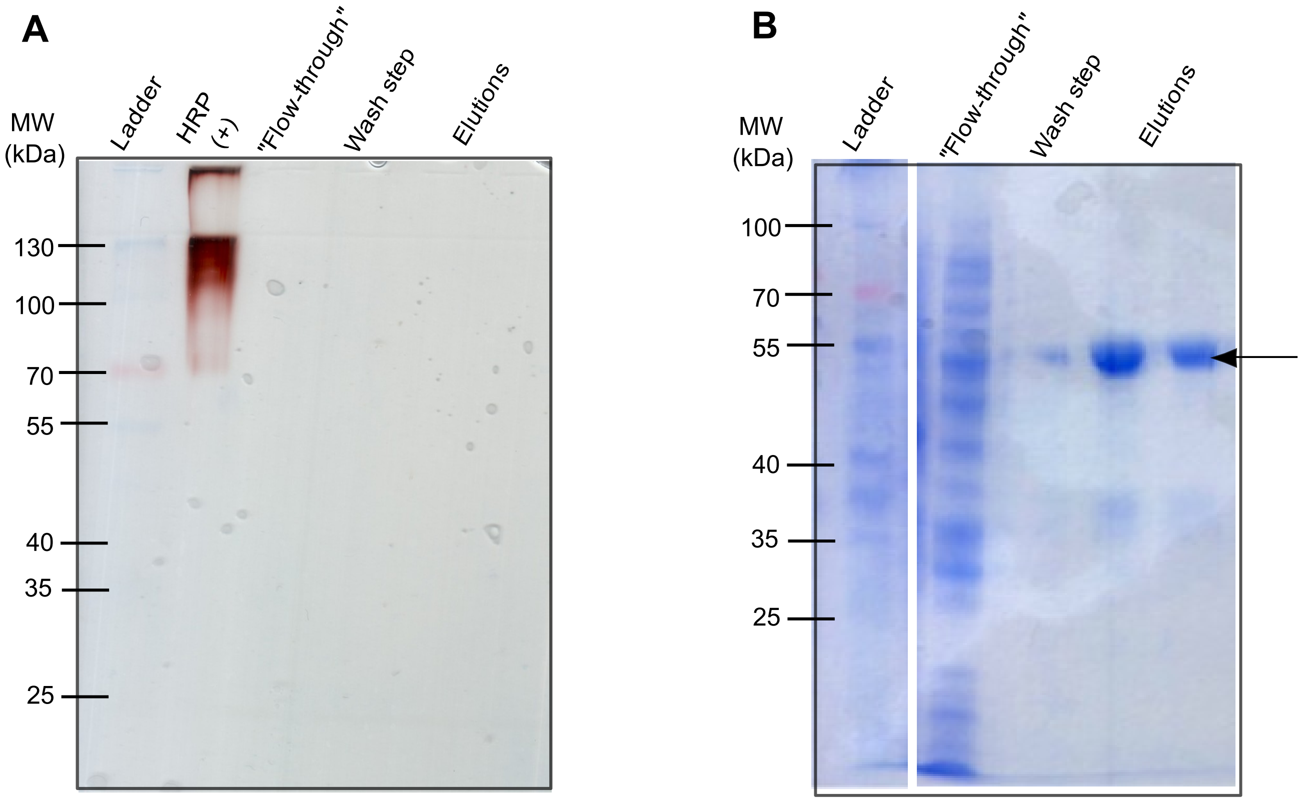

Figure S1 Expression and purification of the recombinant B4FFK9 protein on *E. coli*. A) Guaiacol- H_2O_2 + Coomassie R-250 staining of the fractions obtained by affinity purification. B) Coomassie R-250 staining of the affinity purified fractions. Arrow indicates B4FFK9-GST protein

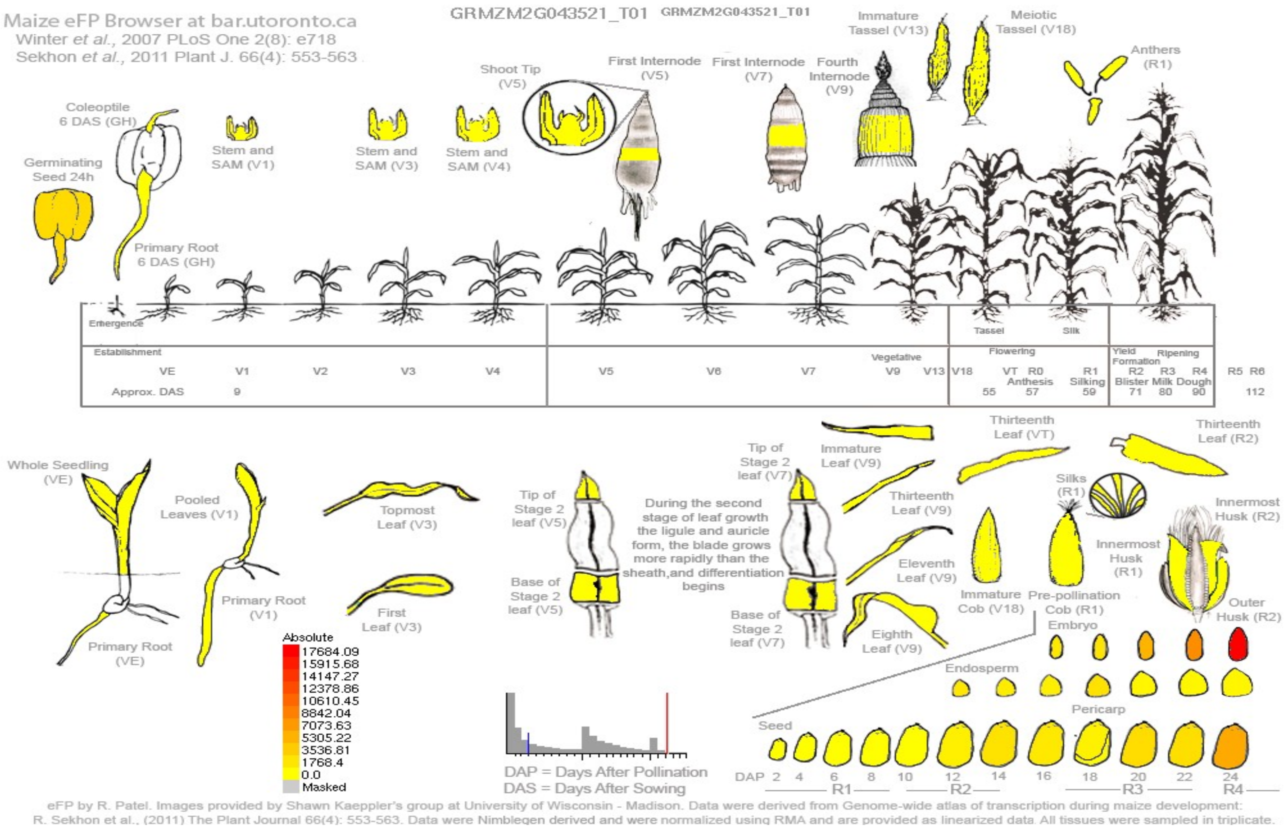

Figure S2. Expression patterns of the accession GRMZM2G043521_T01 accession in B73 maize (corresponding to B4FFK9_MAIZE UniProt accession) using the Maize eFP Browser (http://www.bar.utoronto.ca/).

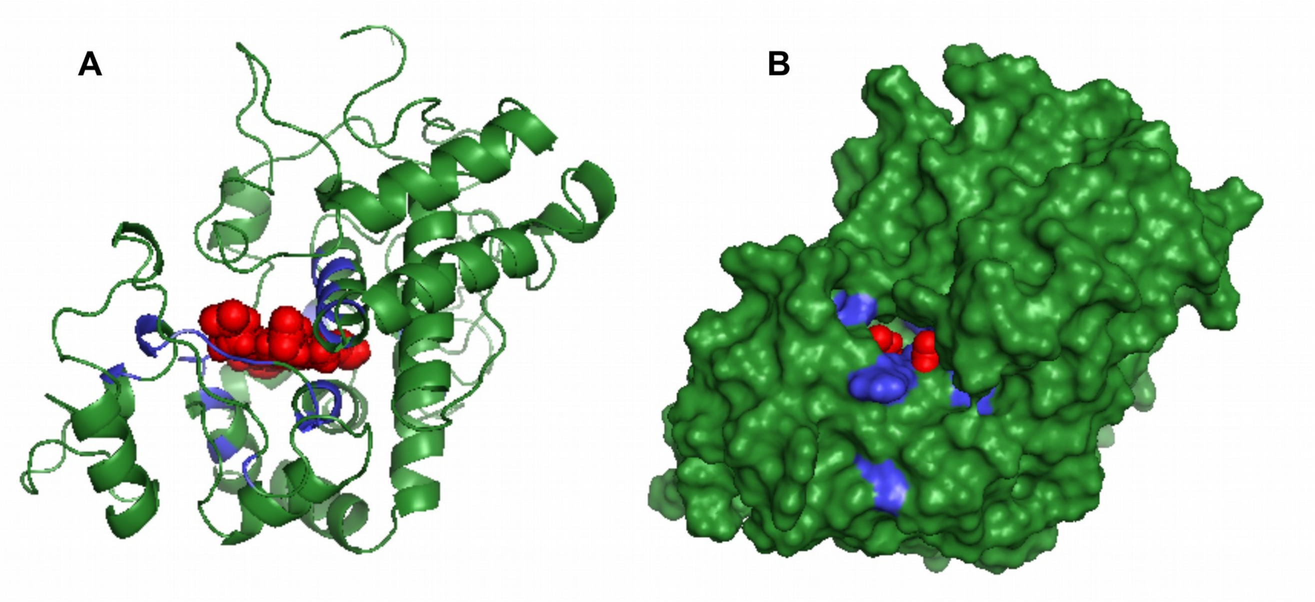

Figure S3 Structural model of B6T173 (ZmPrx35) using the I-TASSER server. A) Cartoon view of the model of ZmPrx35 (green) bound to heme group (red spheres). B) Surface model showing ZmPrx35 (green), bound to heme group (red). The residues predicted to coordinate the heme are shown in blue.

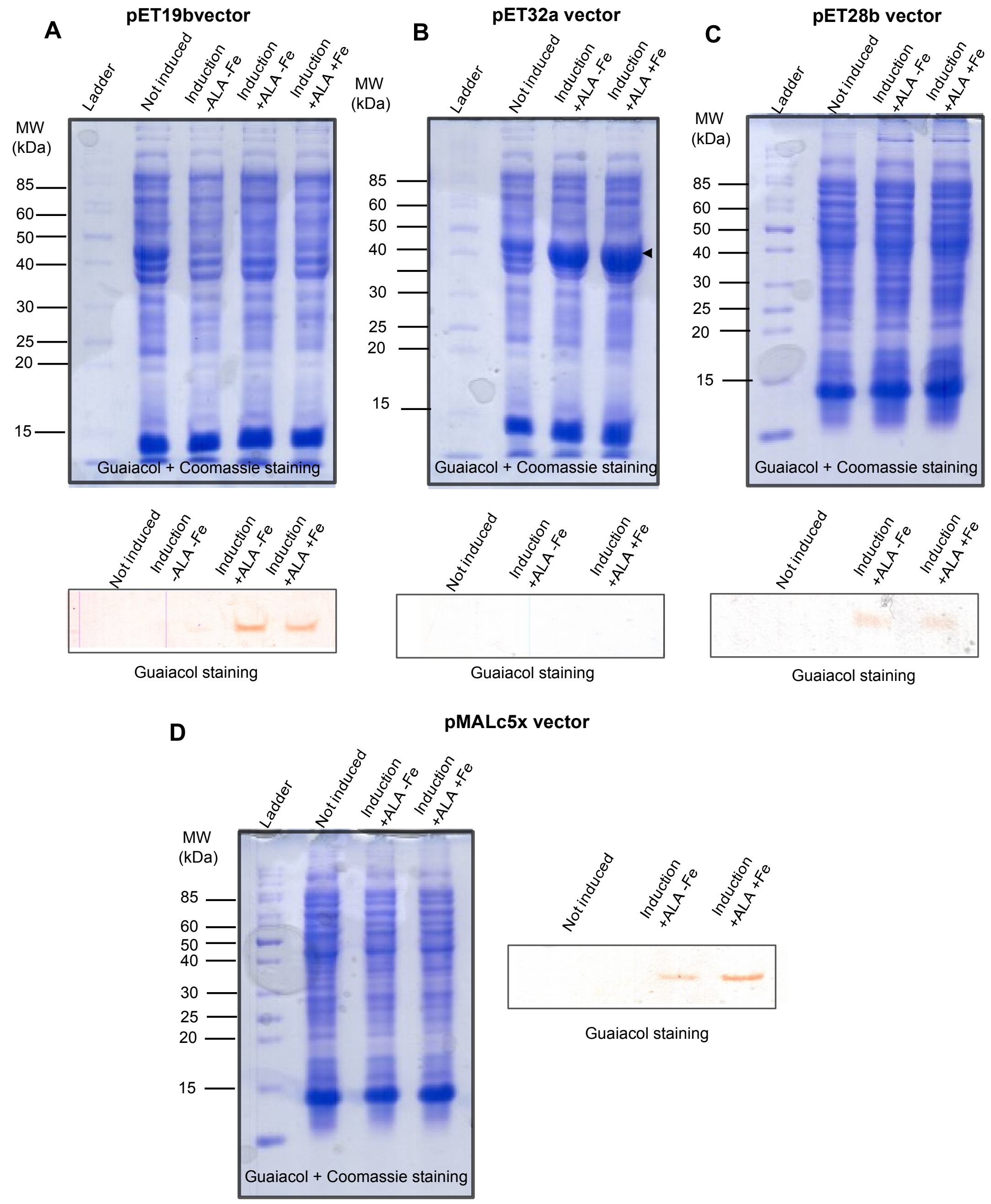

Figure S4 Induction of the recombinant versions of B6T173 (ZmPrx35), using different expression vectors. A) Induction using pET19b vector. B) Induction using pET32a vector and the synthetic version of the gene. C) Induction using pET28b vector. D) Induction using pMALc5x vector, with B6T173 fused to MBP. In all cases, 20 µL of protein of the indicated fraction was loaded on each lane.
